# *O*-GlcNAcylation, an Epigenetic Mark. Focus on the Histone Code, TET Family Proteins, and Polycomb Group Proteins

**DOI:** 10.3389/fendo.2014.00155

**Published:** 2014-09-26

**Authors:** Vanessa Dehennaut, Dominique Leprince, Tony Lefebvre

**Affiliations:** ^1^Structural and Functional Glycobiology Unit, Lille 1 University, Villeneuve d’Ascq, France; ^2^Institut de Biologie de Lille, Pasteur Institute of Lille, Université Lille Nord de France, Lille, France

**Keywords:** *O*-GlcNAcylation, OGT, histones, TET family proteins, polycomb, epigenetic, cancer

## Abstract

There are increasing evidences that dietary components and metabolic disorders affect gene expression through epigenetic mechanisms. These observations support the notion that epigenetic reprograming-linked nutrition is connected to the etiology of metabolic diseases and cancer. During the last 5 years, accumulating data revealed that the nutrient-sensing *O*-GlcNAc glycosylation (*O*-GlcNAcylation) may be pivotal in the modulation of chromatin remodeling and in the regulation of gene expression by being part of the “histone code,” and by identifying OGT (*O*-GlcNAc transferase) as an interacting partner of the TET family proteins of DNA hydroxylases and as a member of the polycomb group proteins. Thus, it is suggested that *O*-GlcNAcylation is a post-translational modification that links nutrition to epigenetic. This review summarizes recent findings about the interplay between *O*-GlcNAcylation and the epigenome and enlightens the contribution of the glycosylation to epigenetic reprograming.

## Introduction

It is widely accepted that cancer is a group of genetic diseases initiated by a sequential acquisition of mutations leading to the constitutive activation of oncogenes and/or the loss of function of tumor suppressor genes. However, numerous studies demonstrated that tumoral development also implies epigenetic modifications, i.e., an alteration of gene expression through mechanisms that do not affect the primary sequence of DNA (1). These epigenetic modifications include perturbations of DNA methylation patterns (repression of tumor suppressor genes and activation of oncogenes by hypermethylation and hypomethylation of their promoter region, respectively) and post-translational modification (PTM) of histone tails that drive chromatin compaction and relaxation that is chromatin dynamics.

An increasing number of studies tend to demonstrate that the “epigenome” is capable of integrating and transmitting nutrient information across generations. For example, the dietary intake of the methyl group donor folate and vitamin B12 to pregnant mice influences the expression of the *Agouti* gene, whose methylation rate defines the color of the coat of the offspring (2). It has also been demonstrated that young mice arisen from mothers undernourished during the pregnancy had defects in the methylation and in the expression of the *leptin* gene encoding a factor controlling satiety and that this phenotype was maintained in adults (3). Therefore, it is obvious that nutrient intake or metabolic disorders could influence the emergence of cancers by modifying the epigenome (4). In this way, a recent study highlighted that, when compared with individuals placed on a normal diet, individuals fed a high lipid diet on a very short period exhibit a modification of the methylome of muscular cells affecting principally genes implicated in the inflammatory response, the reproductive system, and cancer (5). Another recent study also showed that a deprivation in folate led to an increase in the invasive character of human colic cancer cells through hypomethylation of the promoter region of the *Sonic-hedgehog* oncogene and activation of the NF-κB signaling pathway (6). Whereas these two studies among many others lend weight to the hypothesis of a close relationship between nutritional disorders, epigenetic reprograming, and cancer, the underlying mechanisms are still poorly understood. Therefore, the nutrient sensor and chromatin modifier *O*-GlcNAc should be specifically considered as a candidate connecting nutrition to epigenetic and cancer.

## *O*-GlcNAcylation: A Nutrient Sensor Implicated in Cancer Emergence

**Figure 1:**
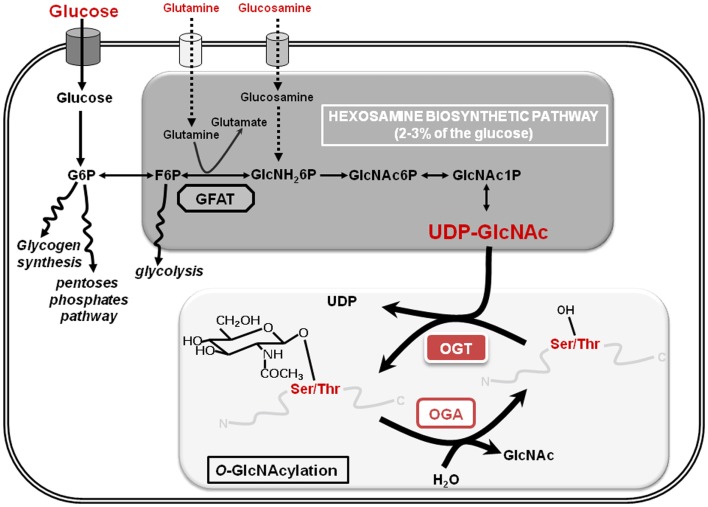
**The hexosamine biosynthetic pathway and *O*-GlcNAcylation are shown**. The hexosamine biosynthetic pathway (HBP) whose key limiting enzyme is GFAT (glutamine:fructose-6-phosphate amido transferase) uses 2–3% of the extracellular glucose to produce UDP-GlcNAc (uridine-di-phospho-*N*-acetyl-glucosamine), the substrate that provides the GlcNAc residue for the *O*-GlcNAcylation processes. This dynamic and reversible post-translational modification of nuclear and cytosolic proteins controls the target proteins fate according to glucose and nutrients availability: it is therefore considered as a nutritional sensor. A single residue of GlcNAc is transferred to a serine or a threonine residue of the protein by the unique *O*-GlcNAc Transferase (OGT) and *O*-GlcNAcase (OGA) hydrolyzes the residue. G6P, glucose-6-phosphate; F6P, fructose-6-phosphate; GlcNH_2_6P, glucosamine-6-phosphate; GlcNAc6P: *N*-acetyl-glucosamine-6-phosphate; GlcNAc1P: *N*-acetyl-glucosamine-1-phosphate.

**Figure 2:**
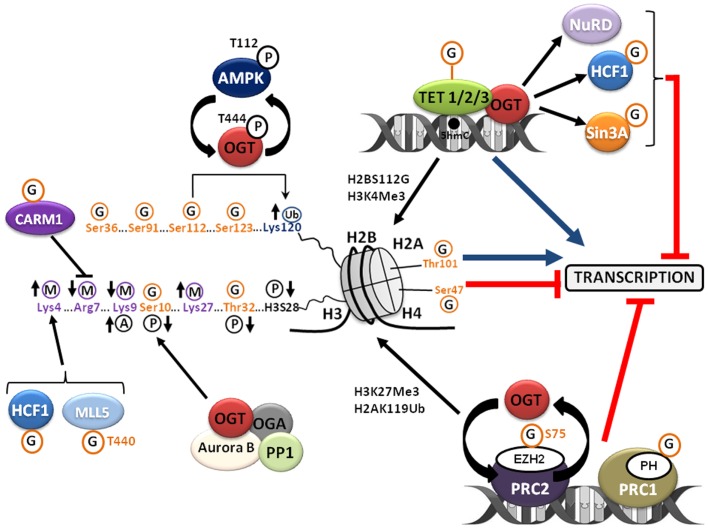
***O*-GlcNAcylation regulates the chromatin dynamics**. *O*-GlcNAcylation regulates chromatin compaction and accordingly gene transcription by interfering with the other post-translational modifications of histones that define the “histone code” and indirectly by its complex relationship with the PcG proteins and the TET family proteins. See the text for details. A, acetylation; G, *O*-GlcNAcylation; M, methylation; P, phosphorylation; Ub, ubiquitination.

*O*-GlcNAcylation or *O*-linked β-d-*N*-acetylglucosaminylation is a reversible PTM of cytosolic, nuclear, and mitochondrial proteins that consists in the covalent linkage of a unique residue of *N*-acetylglucosamine (GlcNAc) to serines and threonines of target proteins. *O*-GlcNAcylation levels are regulated by a unique couple of enzymes: OGT (*O*-GlcNAc transferase) that catalyzes the transfer of GlcNAc from UDP-GlcNAc onto the protein and OGA (*O*-GlcNAcase) that hydrolyzes the residue (Figure 1). *O*-GlcNAcylation levels are closely dependent upon the concentration of UDP-GlcNAc, the second most abundant nucleotide structure in the organism, ATP being the first (40 and 100 nmol/g of tissue, respectively). The glucose, glutamine, fatty acids, uridine, and ATP metabolisms converge on the hexosamine biosynthetic pathway (HBP) to produce the nucleotide-sugar. Thus, UDP-GlcNAc and *O*-GlcNAcylation are considered as sensors of the nutritional state of the organism (7, 8), which can relay the effects of an excessive food supply, malnutrition, obesity, and other metabolic problems that represent high risk factors of cancerization processes (9–11), e.g., overweight and obesity account for more than two-thirds of new cases of type-2 diabetes that in turn doubles colorectal cancer emergence (12). In this way, numerous studies clearly show that *O*-GlcNAcylation plays a significant role in the etiology of cancers at different levels: (i) increased contents of *O*-GlcNAcylation and OGT were characterized in different types of cancer (breast, prostate, colon …); (ii) a modulation of the expression or of the activity of OGT influence the proliferation and/or the invasiveness of cancer cells; (iii) several oncogenic and anti-oncogenic proteins are modified and regulated by *O*-GlcNAc (p53, HIC1, c-myc, FOXM1, NF-κB, β-catenin …); (iv) *O*-GlcNAcylation also participates in the metabolic reprograming of cancer cells. The different aspects of the role of *O*-GlcNAcylation in cancer emergence have been extensively reviewed (13, 14). In recent years, *O*-GlcNAcylation has also emerged as an important regulator of chromatin dynamics since this PTM contributes both to the extensively described chemical modifications of histones (acetylation, methylation, ubiquitination, phosphorylation …) and to DNA methylation patterns that affect chromatin structure. The goal of this review is to summarize recent data showing how *O*-GlcNAcylation is involved in the regulation of the epigenome (Figure 2) and consequently how it could contribute to epigenetic reprograming.

## *O*-GlcNAcylation: A Nutrient Sensor Regulating Chromatin Dynamics

### *O*-GlcNAcylation is part of the “histone code”

The chromatin compaction status governs the accessibility of the transcriptional machinery to DNA and so it has a crucial role in the establishment, the maintenance, and the propagation of gene expression patterns. The nucleosome is the basic unit of chromatin. This is an octamer made of a tetramer of histones H3 and H4 and of two dimers of histones H2A–H2B around which DNA rolls up on 147 bp and is locked by the linker histone H1. The chromatin organization is managed in part by a complex network of PTMs of histones called the “histone code” (15). Histones are methylated, acetylated, phosphorylated, ubiquitinated, ADP-ribosylated, and SUMOylated (Figure 2). These PTMs regulate the interaction of histones with DNA and the ability to recruit chromatin remodeling complexes necessary for transcription, replication, recombination, repair, and mitosis. Several recent studies revealed that the core histones H3–H4–H2A–H2B are also modified with *O*-GlcNAc, adding a supplementary level of complexity to the histone code that is far from being entirely deciphered (16–20). Sakabe and Hart (21) showed that the overexpression of OGT in HeLa cells synchronized in M-phase prevented the increase in histone H3 Ser10 phosphorylation, whereas it attenuated the decrease in H3K9 acetylation and H3K27 trimethylation observed during mitosis (22); these observations point out the crucial role of OGT in modifying histone H3 during mitosis. The same laboratory was the first to report *O*-GlcNAcylation of the four core histones in HeLa cells. *O*-GlcNAcylation levels of histones, especially those of histone H3, decrease during mitosis whereas glycosylation status of histones increases upon heat-shock concomitantly with DNA condensation (16). Three sites of *O*-GlcNAcylation were mapped by MS/MS on histone H2A Thr101, histone H2B Ser36, and histone H4 Ser47, respectively. The *O*-GlcNAcylation of core histones was also reported by Zhang et al. in HEK 293 cells (18). These authors showed that the glycosylation of histones fluctuates all along the cell cycle with a lower level in S-phase. More particularly, histone H3 Ser10 was identified as a site of *O*-GlcNAcylation and, in accordance with Sakabe and Hart findings (21), it was observed that increasing *O*-GlcNAcylation by treating cells with glucosamine was associated with a decreased phosphorylation of histone H3 Ser10. These data demonstrate a direct competition between phosphorylation and *O*-GlcNAcylation at histone H3 Ser10 (18). It was also demonstrated that glucosamine induced an increase of the active epigenetic mark H3K4Me3 concomitantly with a decrease of the repressive mark H3K9Me3. This suggests that *O*-GlcNAcylation influences indirectly the occurrence of other PTMs of histones. The precise function of H3 Ser10 phosphorylation is still currently not fully understood but is clearly associated with regulation of the condensation and/or segregation of chromosomes during mitosis (23). Intriguingly, histone H3 Ser10 phosphorylation is catalyzed by the kinase Aurora B (24) whereas it is dephosphorylated by the phosphatase PP1 (25). These two enzymes physically interact with OGT and OGA in a complex located at the midbody to regulate cytokinesis and exit from mitosis (26). In HeLa cells, Thr32 of histone H3 was highlighted as another site of *O*-GlcNAcylation with a higher level in interphase than in mitosis, which inversely correlated with mitosis-specific phosphorylations on histone H3 (19). The mitosis-specific phosphorylations of histone H3 at Thr32, Ser10 (18), and Ser28 (also phosphorylated by Aurora B) (27) are reduced by treating M-phase synchronized-cells with the two inhibitors of OGA, PUGNAc, or thiamet G, or by overexpressing OGT. Fujiki et al. identified three sites of *O*-GlcNAcylation of histone H2B at Ser91, Ser112, and Ser123 (17). *O*-GlcNAcylation of histone H2B at Ser112 fluctuates in response to extracellular glucose and promotes its monoubiquitination on Lys120 by favoring the interaction of histone H2B with the E3 ubiquitin ligase complex BRE1A/B. In this study, genome-wide analysis revealed that H2B Ser112 *O*-GlcNAcylation was frequently located near transcribed genes, suggesting that histone H2B *O*-GlcNAcylation facilitates gene transcription. A very recent study identified the AMP-activated protein kinase (AMPK) as a regulator of H2B Ser112 *O*-GlcNAcylation (20). AMPK is a sensor of energy status which activity is controlled by the ATP/AMP ratio. AMPK controls cell metabolism and cell growth in response to changes in nutrient availability. AMPK dysfunctions are associated with diseases including diabetes and cancers (28, 29). In their study, Xu et al. demonstrated that activating AMPK with AICAR in mouse embryonic fibroblasts (MEFs) resulted in a decrease in H2B Ser112 *O*-GlcNAcylation and H2B K120 ubiquitination. AMPK directly phosphorylates OGT on Thr444; this phosphorylation does not interfere with OGT activity *per se* on H2B Ser112, but it prevents its loading on chromatin as demonstrated by ChIP experiments. The authors also demonstrated that the catalytic subunit of AMPK, AMPKα1, is *O*-GlcNAcylated and that the knock-down of OGT by RNAi in MEFs led to a decrease in the activating phosphorylation of AMPK on Thr112. As a whole, these results highlight the occurrence of a feedback regulatory loop between OGT and AMPK.

It was also reported that OGT and *O*-GlcNAcylation control the activity of histones methyltransferases (Figure 2). OGT associates with and modifies CARM1 (co-activator associated arginine methyltransferase 1) (21, 30). The overexpression of OGT in HeLa cells decreased the phosphorylation of CARM1 and its methyltransferase activity at Arg7 of histone H3 (H3R7) (21). MLL5, an H3K4 histone methyltransferase and co-activator of RARα, interacts with OGT in a multimeric complex (31). *O*-GlcNAcylation of MLL5 Thr440 potentiates its H3K4 methyltransferase activity and increases granulopoiesis of HL60 promyelocytes in response to retinoic acid (31). Host cell factor 1 (HCF1) is a component of the H3K4 methyltransferase complex SET1/COMPASS (32, 33). *O*-GlcNAcylation of HCF1 enhances the stability of the SET1/COMPASS complex. OGT is necessary for the binding of SETD1A, the component of the complex that bears the methyltransferase activity, to chromatin (33).

In addition to the glucosaminidase activity, OGA exhibits a histone acetyl transferase (HAT) property in its C-terminal region and is sometimes called NCOAT for nuclear and cytoplasmic *O*-GlcNAcase and acetyl transferase (34). Therefore, *O*-GlcNAcylation would be able to regulate the acetylation of the histones tails, but this function remains controversial (35). Whisenhunt et al. demonstrated that OGA/NCOAT, OGT, the co-repressor Sin3A (Switch-independent 3A), and HDAC1 (Histone Deacetylase 1) co-exist in a complex that was named *O*-GlcNAczyme (36). ChIP experiments performed in MCF7 cells revealed a specific enrichment of the *O*-GlcNAczyme on promoters of repressed genes (36). Nevertheless, the existence of this *O*-GlcNAczyme complex has not yet been confirmed by other studies.

### *O*-GlcNAcylation modifies members of the TET family proteins

In 2013, several studies, albeit sometimes contradictory, provided compelling evidences of a close relationship among OGT, *O*-GlcNAcylation, and the DNA hydroxylase properties of the TET (Ten–Eleven Transcription) family proteins involved in the DNA demethylation on CpG islands (Figure 2). TET1, TET2, and TET3 convert 5-methyl-cytosine (5mC) to 5-hydroxy-methyl-cytosine (5hmC) and are necessary for gene transcription, pre-mRNA splicing, and zygotic genetic reprograming (37). Chen et al. demonstrated that OGT interacts but does not *O*-GlcNAcylate or influence the function of TET2 and TET3 (38). However, these authors showed that TET2 and TET3 promote the recruitment of OGT to the chromatin in order to modify histones (38). Such a role for TET3 in the loading of OGT to chromatin has been also reported by Ito et al. at the same time (39). In their study, Chen et al. performed ChIP-Seq experiments that revealed the presence of OGT and H2B Ser112-*O*-GlcNAc on a large number of TET2 target genes and more especially around transcription start sites (38). Contrary to the observations of Chen et al., *O*-GlcNAcylation of transiently transfected TET3 was reported in two other studies, but *O*-GlcNAcylation of the endogenous protein was not observed (39, 40). The *O*-GlcNAcylation of TET1 and TET2 were also independently reported (40–42). OGT promotes the cytoplasmic relocation of a myc-TET3 construction according to a mechanism that remains to be deciphered whereas *O*-GlcNAcylation has no effect either on TET1 or on TET2 subcellular localization (40). However, *O*-GlcNAcylation of TET1 regulates its expression level in mouse embryonic stem (ES) cells (42) and stabilizes the hydroxylase on the promoters of target genes (41). The occurrence that OGT and TET proteins form a complex with co-repressors reinforces the importance of *O*-GlcNAcylation processes in the regulation of gene transcription. By proteomic analyses performed in mouse ES cells, Shi et al. demonstrated that TET1 and OGT interact with the chromatin regulator Sin3A and with several members of the NuRD (nucleosome remodeling and deacetylase) complex (Figure 2) (40). The authors also found that OGT is required for maintaining ES cells pluripotency since depletion of this enzyme induces a derepression of several markers of differentiation (40). The interaction between TET1, TET2, Sin3A, HCF1, and OGT was also reported (41). In this study, using genome-wide ChIP-seq experiments, 11552 binding sites for OGT among which 62% are located within promoter regions were identified. A co-localization of OGT, TET1, and H3K4Me3 was also observed near the transcription start sites; this demonstrates that TET1 is necessary to recruit OGT to the chromatin.

### OGT belongs to the polycomb group proteins

In 2009, two independent studies surprisingly revealed that the fly gene *Sxc* (*Supersexcomb*) initially characterized as a gene belonging to the polycomb group (PcG) proteins (43) is the gene that encodes OGT (44, 45). PcG proteins represent a family of transcriptional repressors discovered in *Drosophila melanogaster*. These proteins are necessary for the maintenance of the repression of the homeotic genes (*Hox*) whose expression patterns govern the establishment of the antero-posterior axis of the embryo. In mammals, PcG proteins also repressed *Hox* genes and numerous other genes controlling the programing of adult and ES cells, cell proliferation, and differentiation. In *Drosophila* and mammals, PcG proteins are found in two main large complexes, PRC1 and PRC2 (Polycomb Repressive Complexes 1 and 2), whose members have been conserved during evolution (46). These two complexes act in a sequential manner: first PRC2 is recruited to the promoter region of its target genes where the histone methyl transferase EZH2 (Enhancer of Zest Homolog 2) is responsible for H3K27 di- and trimethylation (H3K27Me2 and H3K27Me3), a repressive epigenetic mark. H3K27Me3 is subsequently recognized by the chromodomain of the PC (Polycomb) protein, a core component of PRC1. The catalytic activity of PRC1 is driven by the E3 ubiquitin ligase RING that catalyzes the monoubiquitination of histone H2A Lys119 (H2AK119Ub). The precise mechanisms by which Polycomb complexes repress transcription are not fully understood while it involves both inhibition of the transcriptional machinery and chromatin compaction and recruitment of DNA methyl transferases (DNMTs). Polycomb responsive elements (PRE) were characterized in *Drosophila*, but such nucleotide sequences are not found in mammals. Therefore, in mammals, the mechanisms of PRC2 recruitment to its target genes remain to be elucidated. One proposed targeting mechanisms together with sub-stoichiometric components of PRC2 (e.g., JRID2) or the implication of specific long non-coding RNAs in the interaction of PRC2 components with sequence-specific transcription factors (47). For example, the transcriptional repressor HIC1 (Hypermethylated in cancer 1) could recruit PRC2 on a subset of target genes through its interaction with human polycomb-like (hPCL) proteins (48). For note, we previously demonstrated that HIC1 is *O*-GlcNAcylated, but the role of its glycosylation is still not deciphered (49). In their original study performed in *Drosophila*, Gambetta and collaborators identified by ChIP-seq experiments 1138 sites occupied by *O*-GlcNAcylated proteins among which 490 colocalized with PREs (44). The authors demonstrated a decrease of the fixation of the polyhomeotic (PH) protein, a core component of PRC1, on the majority of the PREs in *sxc/ogt* mutants in comparison with wild-type *Drosophila*. The same authors also showed that PH is itself *O*-GlcNAcylated (Figure 2). The catalytic core component of PRC2 EZH2 interacts with OGT inside PRC2 and the knock-down of OGT in MCF7 or MDA-MB231 cells led to a 50% decrease in H3K27Me3 due to EZH2 and PRC2 destabilization (50). *O*-GlcNAcylation of EZH2 has been mapped at Ser75, and the S75A mutant is less stable than the wild-type protein (Figure 2). By a combination of microarrays and ChIP experiments, Chu et al. (50) identified 16 genes co-regulated by OGT and EZH2. Knock-down of OGT affects the fixation of EZH2 and the deposit of the repressive mark H3K27Me3 on these genes, some of which like *UNC5A* or *IL1R1* have been reported as tumor suppressor genes (51, 52). A study conducted in mouse highlighted a decrease in OGT and in nuclear *O*-GlcNAcylation in *eed*^−/−^ and *suz12*^−/−^ ES cells, two genes encoding core components of PRC2 (53). This set of data suggests a complex feedback relationship between *O*-GlcNAcylation and PcG proteins, notably with core components of PRC2, which remains to be fully understood.

## Conclusion and Future Directions

*O*-GlcNAcylation has recently emerged as a novel epigenetic mark affecting chromatin remodeling and gene expression according to several mechanisms (Figure 2). First, *O*-GlcNAcylation modifies histone tails and depending on the residue, *O*-GlcNAcylation either favors chromatin relaxation and gene transcription or chromatin compaction and thus it prevents transcription. *O*-GlcNAcylation also regulates the occurrence of other PTMs defining the histone code and more particularly methylation by modulating the activity of several methyltransferases like CARM1, MLL5, and HCF1. OGT and *O*-GlcNAcylation regulates the activity of different co-repressors among which NuRD and mSin3A; but, especially, *O*-GlcNAcylation displays a complex relationship with the PcG proteins to prevent gene transcription. At last, *O*-GlcNAcylation may promote DNA demethylation by interacting with members of the TET family proteins thus favoring gene transcription.

At this time, the crucial role played by *O*-GlcNAcylation in metabolic disorders and neuronal diseases etiology is indisputable. Regarding cancer, *O*-GlcNAcylation interfered with cell biology through a large panel of mechanisms among cell proliferation, adhesion, migration, and metabolic reprograming. In this review, we summarized recent evidences suggesting that *O*-GlcNAcylation also highly coordinates chromatin dynamics adding a further level of regulation of cancer emergence through *O*-GlcNAcylation of the epigenome. However, the role of *O*-GlcNAcylation in cancer-associated epigenetic reprograming is far from being fully deciphered and further studies are required to understand the impact of aberrant *O*-GlcNAcylation in tumorigenesis and to identify new targets that could be used for prevention, diagnosis, or treatment of cancers.

## Conflict of Interest Statement

The authors declare that the research was conducted in the absence of any commercial or financial relationships that could be construed as a potential conflict of interest.
